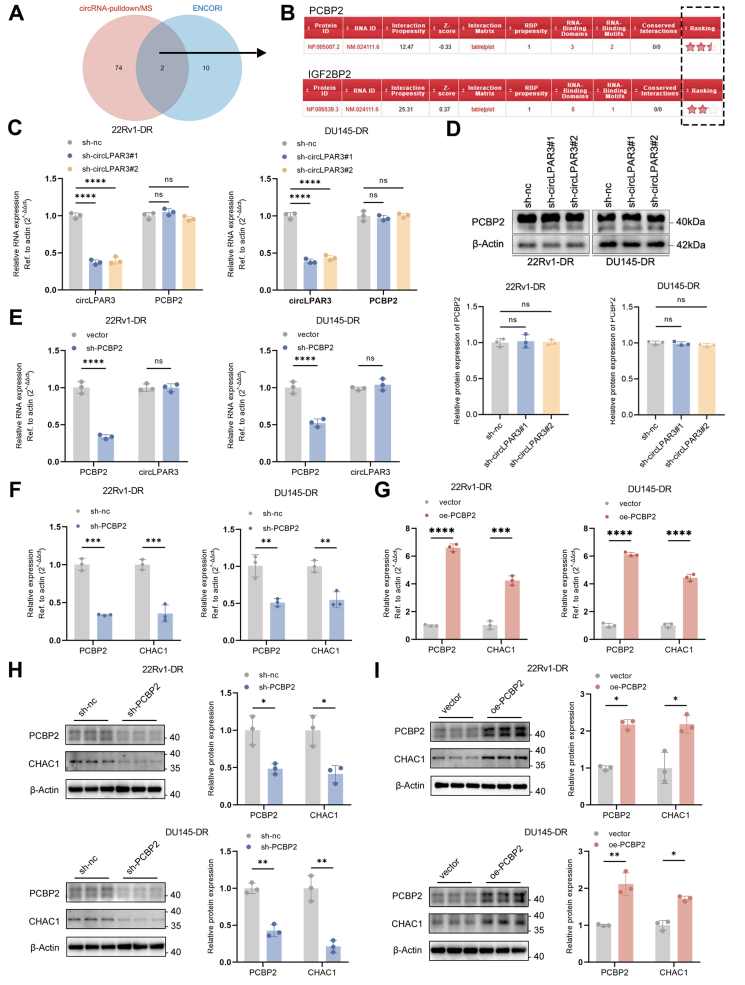# N6-methyladenosine-modified circLPAR3 drives docetaxel resistance in prostate cancer by suppressing ferroptosis through the PCBP2/CHAC1 axis

**DOI:** 10.1016/j.gendis.2025.101816

**Published:** 2025-08-22

**Authors:** Yetao Zhang, Yuxiang Dong, Kai Li, Tong Zhao, Yongshan Li, Mingyang Pang, Yong Wei, Bing Yao, Qingyi Zhu

**Affiliations:** a Department of Urology, The Second Affiliated Hospital of Nanjing Medical University, Nanjing, Jiangsu 210011, China; b Department of Medical Genetics, Nanjing Medical University, Nanjing, Jiangsu 211166, China

**Keywords:** CHAC1, Chemoresistance, circLPAR3, Docetaxel, Ferroptosis, N6-methyladenosine, Prostate cancer

## Abstract

Epigenetic mechanisms represented by N6-methyladenosine (m^6^A) modifications and circular RNAs (circRNAs) regulate chemotherapy resistance in prostate cancer (PCa). Here, we stuided the role of m^6^A-modified circLPAR3 in assisting docetaxel (DTX) resistance in PCa and the mechanism of its regulatory function. The expression of circLPAR3 was higher in all three drug-resistant cell lines than in the corresponding parental cell lines and was mainly localized in the cytoplasm. The m^6^A modification and stability enhancement of circLPAR3 by the METTL3/IGF2BP2 axis was primarily determined by expression assays, stability assays, and mass spectrometry. Functionally, circLPAR3 reduced the sensitivity of PCa cells to DTX and promoted PCa proliferation in a DTX environment. In addition, circLPAR3 reduced cytotoxicity by inhibiting the DTX-induced ferroptosis effect. Mechanistically, circLPAR3 downregulated the expression of CHAC1, an emerging driver of ferroptosis. PCBP2 was identified as a mediating RNA-binding protein between circLPAR3 and CHAC1. On the one hand, it shares a very high binding phase with circLPAR3 without interfering with each other’s expression; on the other hand, it increases mRNA stability by binding to the 3′ UTR of CHAC1, which is interfered by circLPAR3. In conclusion, m^6^A-modified circLPAR3 inhibits CHAC1 expression by competitively binding to PCBP2, ultimately conferring docetaxel resistance to PCa.

## Introduction

Prostate cancer (PCa) ranks as one of the most prevalent malignancies and is the second leading cause of cancer-related deaths in men, with an estimated 288,300 new cases and 34,700 deaths in the United States in 2023.^1^ Early diagnosis and intervention improve clinical outcomes; however, advanced recurrence and metastasis remain significant challenges. While second-generation anti-androgen therapies offer clinical benefits, their prognosis and side effects remain suboptimal.^2^ Docetaxel (DTX) has proven effective in the treatment of metastatic castration-resistant prostate cancer (mCRPC), yielding notable clinical benefits.^3^, ^4^, ^5^ However, the emergence of DTX resistance is a primary cause of treatment failure. The development of such resistance is a multifactorial process driven by various epigenetic mechanisms.^6^ Therefore, understanding the molecular mechanisms underlying DTX resistance is crucial for improving patient prognosis.

Research into the functional roles of circular RNAs (circRNAs), a unique family of non-coding RNAs, in cancer has advanced significantly. It has been demonstrated that circRNAs can directly or indirectly regulate proteins, RNA, and DNA, thereby influencing tumor resistance to DTX in PCa. For instance, circARHGAP29 mediates DTX resistance in PCa through the IGF2BP2/c-Myc/LDHA pathway;^7^ circCYP24A1 promotes resistance by upregulating ALDH1A3;^8^ and the circ_0004087/SND1/MYB/BUB1 axis affects the mitotic error correction mechanism, regulating DTX resistance.^9^ Moreover, in the progression and treatment resistance of various cancers, methylation-related enzymes mediate the methylation modification of circRNAs, stabilizing their expression in tumors and enhancing their epigenetic regulatory roles. Several methylated circRNAs, such as circFLIS3, circRBM33, and circDDIT4, have been implicated in PCa progression.^10^, ^11^, ^12^, ^13^, ^14^, ^15^ However, research focusing on methylated circRNAs in DTX resistance in PCa remains limited and warrants further investigation.

Ferroptosis is an iron-dependent form of programmed cell death characterized by the accumulation of reactive oxygen species (ROS) and lipid peroxidation. Recent studies have demonstrated that the induction or inhibition of ferroptosis in tumor cells can modulate the sensitivity of PCa cells to DTX. Our previous work has shown that DTX induces ferroptosis in PCa cells.^16^ Additionally, Sun et al have proposed that miR-432-5p secreted by tumor-associated fibroblasts promotes DTX resistance in PCa by inhibiting ferroptosis,^17^ while Zhang’s study has revealed that lncRNA PCAT1 suppresses ferroptosis in DTX-resistant PCa cells via a ceRNA mechanism.^18^ However, the role of methylated circRNAs in mediating chemotherapy resistance through ferroptosis regulation remains unclear.

This study utilized multiple sequencing datasets, identifying circLPAR3 as a potential mediator of DTX resistance in PCa. A series of phenotypic validations and mechanistic investigations demonstrated that circLPAR3 is upregulated in DTX-resistant PCa cells and undergoes m6A modification mediated by insulin-like growth factor 2 mRNA-binding protein 2 (IGF2BP2). Functional experiments demonstrated that circLPAR3 promotes the growth of DTX-resistant PCa cells both *in vitro* and *in vivo*, while also protecting against ferroptosis. Mechanistically, circLPAR3 mitigates the ferroptosis induced by DTX by disrupting the stabilizing effect of Poly(RC) Binding Protein 2 (PCBP2) on the mRNA of ChaC Glutathione Specific Gamma-Glutamylcyclotransferase 1 (CHAC1) in the cytoplasm. This study enhances our understanding of the role of methylated circRNAs in chemotherapy resistance in PCa and offers a novel strategy to improve the sensitivity of PCa to chemotherapy.

## Methods and materials

### Cell culture

Human PCa cell lines (22Rv1, DU145) were obtained from Wuhan Procell Life Science and Technology Co., Ltd., China. All cell lines underwent short tandem repeat analysis and mycoplasma testing (Novoprotein). The 22Rv1 cells were cultured in RPMI-1640 medium (WISENT, Nanjing) supplemented with 10% fetal bovine serum (FBS) (WISENT, Nanjing), while DU145 cells were cultured in Dulbecco's Modified Eagle Medium (WISENT, Nanjing). All cells were maintained in a 37 °C incubator with 5 % CO2. Cell morphology was monitored daily, the medium was changed every 2–3 days, and cells were passaged when confluence reached 90%. All cultures and plating were performed in tissue culture-treated cell culture flasks and other appropriate vessels.

DTX-resistant 22Rv1 and DU145 cell lines were established via a drug concentration escalation method. Cells with 80% confluence were treated with a complete medium containing DTX for 48 h. The culture medium was discarded, and cells were washed three times with phosphate-buffered saline (PBS) before being replaced with a complete medium without the drug. Once the cells recovered to 80% confluence, the medium containing DTX was reintroduced. The drug concentration was progressively increased after each treatment cycle, which was repeated six times. The IC50 of the induced cells was determined, and cells with an IC50 ratio (induced/parent) > 5 were considered resistant.

### RNA purification, reverse transcription, and quantitative PCR

Total RNA extraction and purification were performed using FreeZol Reagent (Vazyme Biotech Co., Ltd., Nanjing, China). cDNA synthesis was conducted with HiScript III All-in-one RT SuperMix Perfect for quantitative PCR (Vazyme Biotech), using random hexamers for circRNA and oligo(dT)23 primers for mRNA. Quantitative PCR was carried out with ChamQ SYBR qPCR Master Mix (Without ROX) (Vazyme Biotech) on the LightCycler® 480 System (Roche). Relative expression was calculated using the 2^−ΔΔCt^ method and normalized to β-actin. All primer sequences used in this study were synthesized by Tsingke Biotechnology and listed in Supplementary Table 1.

### Plasmid construction and cell transfection

The lentiviruses used for hsa_circ_0004390 knockdown and overexpression and the corresponding controls were purchased from Tsingke (Nanjing, China). PCa cells were infected with lentivirus following the manufacturer’s instructions. The full-length complementary cDNA of human PCBP2 and CHAC1 was synthesized, subcloned, and then inserted into the PLV3-CMV expression vector by Corues Biotechnology (Nanjing, China). Cell transfection assays were conducted using Lipomaster 3000 Transfection Reagent (Vazyme Biotech, Nanjing, China). ShRNAs used are shown in Supplementary Table 2.

### circRNA pull-down, silver staining, and mass spectrometry

Biotin-labeled probes targeting the junction of circLPAR3 were synthesized by GenePharma. RNA pull-down was performed using the PureBinding® RNA-Protein Pull-Down Kit (Geneseed Biotech Co., Ltd., Guangdong, China) according to the manufacturer’s instructions. The biotinylated probe was incubated with streptavidin-coated magnetic beads, followed by incubation with cell lysate to form a complex. RNA-binding proteins (RBPs) were eluted from the bioconjugated complex, and further analyses were conducted using Western blotting and mass spectrometry (MS). The probes used in the study are listed in Supplementary Table 3. Silver staining of the samples was performed with the Fast Silver Stain Kit (Beyotime). Differences between the sense and anti-sense groups were assessed based on the intensity of the silver stain. The corresponding gel blocks were collected from the SDS-PAGE gel after Western blotting. MS analysis was performed by Shanghai IProteome Biotechnology Co., Ltd., with protein identification and quantification completed via the company’s cloud platform. Differentially expressed proteins were identified by comparing the two groups, and the binding reliability of these proteins to circRNA was assessed based on the iBAQ (absolute protein level) value and the number of unique peptides.

### RNA immunoprecipitation

RNA immunoprecipitation (RIP) assays were conducted using the PureBinding® RNA Immunoprecipitation Kit (Geneseed Biotech) according to the manufacturer’s instructions. Briefly, 5 μg of antibodies against IGF2BP2, PCBP2, or species-specific IgG were pre-coated onto magnetic beads. The antibody–bead complex was incubated with cell lysates overnight, followed by RNA elution and purification. Reverse transcription and quantitative PCR were subsequently performed on the purified RNA. Detailed antibody information is provided in Supplementary Table 4.

### RNA sequencing

RNA sequencing (RNA-seq) was performed on circLPAR3 knockdown cells (si-circLPAR3#1) and negative control (si-negative control) 22Rv1-DR cells. Total RNA was extracted using FreeZol Reagent (Vazyme Biotech). Sequencing was carried out by Majorbio Technology using the Illumina platform, followed by differential analysis and functional annotation on their cloud platform. Differentially expressed genes (DEGs) were identified using the criteria of adjusted *P* < 0.05 and fold change > 2.

### Western blotting

Protein expression of IGF2BP2, PCBP2, and CHAC1 was assessed by Western blotting. Total protein was extracted from cells using a cell lysis buffer for Western blotting and Co-immunoprecipitation (COIP) with inhibitors (Affinibody LifeScience Co., Ltd., Switzerland). The membrane was blocked with 5% skimmed milk at room temperature for 2 h and then incubated overnight at 4 °C with primary antibodies. The following day, the membrane was washed and incubated with horseradish peroxidase-conjugated secondary antibodies (anti-rabbit or anti-mouse, 1:5000) for 1 h at room temperature. Protein bands were visualized using a gel imaging system (Tanon) and quantified with ImageJ software. Antibodies and dilution ratios are listed in Supplementary Table 4.

### Cell counting kit-8 (CCK-8) assay

For cell proliferation or viability assessment, PCa cell suspensions (100 μL/well) were seeded in 96-well plates and treated as indicated. Cell proliferation was measured using the Cell Counting Kit-8 (CCK-8) (APExBIO, USA) following the manufacturer’s protocol. CCK-8 solution (10 μL) was added to each well and incubated at 37 °C for 4 h (22Rv1 and 22Rv1-DR) or 2 h (DU145 and DU145-DR). Absorbance at 450 nm was measured using a microplate reader.

### Colony formation assay

Approximately 1000 cells were seeded in 6-well plates and cultured at 37 °C with 5% CO2 for 2 weeks. After removing the medium, cells were washed with PBS, fixed with 4% paraformaldehyde (Beyotime, China), and stained with 1% crystal violet (Beyotime, China). Colonies were imaged using a flatbed scanner and counted using ImageJ software.

### EdU assay

For cell inoculation, 24-well plates were used, and the cells were cultured in a 37 °C incubator with 5% CO2 for 24 h before transfection and drug treatment. Following the treatments, EdU detection was carried out using the BeyoClick™ EdU-594 Cell Proliferation Detection Kit (Beyotime, China). The cells were incubated with 10 μM EdU reagent for 2 h. After incubation, cells were fixed with 4% paraformaldehyde and permeabilized with 0.5% Triton X-100. The detection working solution (200 μL) was added to each well and incubated in the dark for 30 min. Nuclei were stained with 1 × Hoechst 33342, and the samples were visualized using the Thunder Panoramic Tissue Microscopy Imaging System (Leica, China). ImageJ was used to calculate the number of EdU-positive cells relative to the total number of cells.

### Cytotoxicity assay

Cytotoxicity was evaluated by measuring lactate dehydrogenase (LDH) release using the Cytotoxicity Detection Kit (LDH) (Beyotime Biotech, China). After the specified treatment, 100 μL of cell suspension was added to the 96-well culture plate. Following centrifugation at 400 *g* for 5 min, 120 μL of the supernatant was collected, and 60 μL of the LDH detection working solution was added to each sample. The samples were incubated in the dark at room temperature for 30 min, and absorbance at 490 nm was measured to determine cytotoxicity.

### Cellular ROS detection

To detect intracellular ROS levels, the ROS assay kit (Beyotime, China) was used. Cells were rinsed twice with PBS and incubated with a DCFH-DA probe (diluted in basal medium) for 20 min in the dark at 37 °C. Fluorescence intensity (Ex: 488 nm; Em: 525 nm) was measured using the Synergy Lx Multimode Reader (Agilent, USA).

### Lipid ROS assay

Lipid peroxidation levels were assessed using BODIPY™ 581/591 C11 (ThermoFisher Scientific, USA). After treatment, cells were digested, collected, and rinsed with Hank's balanced salt solution (HBSS). They were then resuspended in 1 mL basal medium containing 0.1% BODIPY-C11 and incubated in the dark at 37 °C for 30 min. Excess medium was removed, and cells were washed twice with HBSS and then resuspended in HBSS. Flow cytometry was performed using the BD FACSCanto™ II (BD, USA), and at least 10,000 cells were analyzed per sample to determine the fluorescence intensity of lipid peroxidation.

### Malondialdehyde (MDA) assay

The MDA concentration in the cell lysate was quantified using the MDA assay kit (Beyotime, China) according to the manufacturer’s instructions. First, total cell protein was extracted and quantified using the BCA Protein Concentration Assay Kit (Abbkine Scientific Co., Ltd., Georgia, USA). Subsequently, 100 μL of the supernatant from each homogenized sample was transferred to a microcentrifuge tube, followed by the addition of 200 μL of detection working solution. After thorough mixing, the mixture was incubated at 100 °C for 30 min, cooled to room temperature in a water bath, and centrifuged at 1000 *g* for 10 min. A 200 μL aliquot of the supernatant from each tube was transferred to a 96-well plate, and absorbance at 532 nm was measured using the Synergy Lx Multimode Reader.

### GSH assay

Intracellular reduced glutathione (GSH) levels were measured using the GSH and oxidized glutathione (GSSG) detection kit (Beyotime, China). After processing the samples as per the kit instructions, absorbance at 412 nm was measured to determine the total glutathione and GSSG concentrations. GSH content was then calculated using the formula: GSH = Total Glutathione − GSSG × 2.

### Iron detection assay

Intracellular ferrous (Fe^2+^) levels were assessed using the FerroOrange probe (Dojindo, Japan) following the manufacturer’s protocol. Cells were cultured and treated in a clear-bottom black 96-well plate (Beyotime, China), washed three times with HBSS, and incubated with 100 μL of FerroOrange working solution for 30 min at 37 °C. Fluorescence intensity (Ex: 543 nm; Em: 580 nm) was measured using a BioTek Synergy H1 microplate reader (USA).

### Xenograft mouse model and drug administration

Six-week-old male BALB/c nude mice from the Animal Center of Nanjing Medical University were selected for the experiment, with five mice per group. Following anesthesia, 8 × 106 lentivirus-infected or negative control 22Rv1 cells and 22Rv1-DR cells were subcutaneously injected into each mouse. From the 10th day onwards, tumor-bearing mice were administered DTX (5 μg/g) twice weekly. Tumor size was measured every five days using a ruler. On day 25, the mice were anesthetized and sacrificed, and their subcutaneous tumors were excised, weighed, and photographed for subsequent immunohistochemical analysis and other assays.

### Statistical analysis

Statistical analysis was conducted using GraphPad Prism 9.5.1. Data are expressed as the mean ± standard deviation or mean ± standard error of the mean from at least three independent experiments. Student’s *t*-test, one-way, or two-way ANOVA was used to assess group differences, with *P* < 0.05 considered statistically significant.

## Results

### Screening and characteristics of circLPAR3 in PCa

To investigate the role of m^6^A-modified circRNAs in DTX-resistant PCa, sequencing data were integrated with publicly available data.^19^, ^20^, ^21^, ^22^ The Venn diagram illustrates the differentially expressed m^6^A-modified circRNAs in both parental and resistant PCa cell lines (Fig. 1A). The differential expression of circRNAs was further validated by quantitative reverse transcription PCR (qRT-PCR) in induced-resistant and parental cells. Among the three cell lines, hsa_circ_0004390 exhibited elevated expression in all resistant cell lines (Fig. 1B), whereas the expression of hsa_circ_0004182 varied across the cell lines (Fig. S1A). Consequently, hsa_circ_0004390 was selected as the focal molecule in this study. Hsa_circ_0004390 (chr12:69644908-69656342) is derived from the linear gene LPAR3 (referred to as circLPAR3), with a length of 754 bp and a single-exon structure formed by exon 2. The circLPAR3 sequence was confirmed by Sanger sequencing at the junction site (Fig. 1C). The head-to-tail splicing nature of circLPAR3 was further validated by qRT-PCR using divergent and convergent primers (Fig. 1D). To assess the stability of circLPAR3, resistant PCa cells were treated with ribonuclease R (RNase R), an enzyme that degrades linear RNA. In contrast to the parental gene, circLPAR3 exhibited marked resistance to RNase R digestion, confirming the stability of its circular structure (Fig. 1E). Additionally, RNA decay assays demonstrated that circLPAR3 degraded at a slower rate than the linear LPAR3 transcript (Fig. 1F). To determine the cellular localization of circLPAR3, RNA cytoplasmic/nuclear fractionation was performed in 22Rv1-DR and DU145-DR cells, followed by qRT-PCR analysis. The results revealed that the majority of circLPAR3 was localized to the cytoplasm (Fig. 1G), a finding further supported by fluorescence *in situ* hybridization (FISH) detection (Fig. 1H). In addition, relevant expression levels were also verified for the parent gene LPAR3 of circLPAR3. The results of both qRT-PCR and Western blotting showed that its parent gene LPAR3 did not show significant expression differences between the drug-resistant line and the parent cell line (Fig.S1B, S1C). Thus, circLPAR3 may be a drug resistance-associated molecule with independent biological functions.

**Figure 1:**
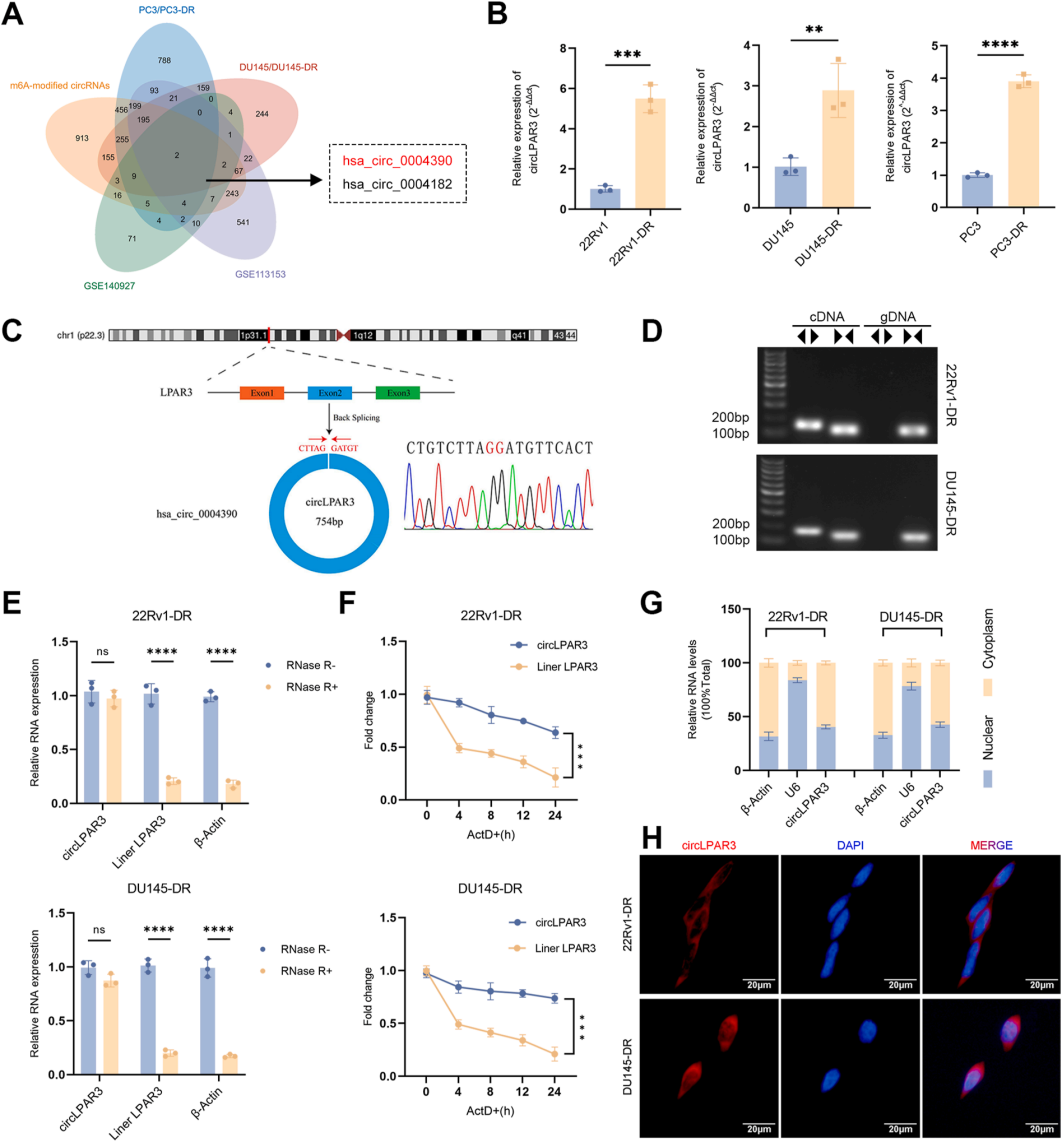
circLPAR3 is verified and characterized in docetaxel-resistant prostate cancer cells. **(A)** Venn plot of the differentially expressed docetaxel-resistant circRNAs. **(B)** The expression of circLPAR3 was detected by quantitative reverse transcription PCR in docetaxel-resistant prostate cancer cells (22Rv1-DR, DU145-DR, and PC3-DR) and sensitive cell lines (22Rv1, DU145, and PC3). **(C)** Schematic illustration of circLPAR3 formation via the circularization from exon 2 in the LPAR3 gene. **(D)** The expression of circLPAR3 in cDNA and gDNA of 22Rv1 and DU145 cells were confirmed by quantitative reverse transcription PCR with the divergent and convergent primers. **(E)** The relative expression of circLPAR3 and LPAR3 was measured by quantitative reverse transcription PCR after treatment with RNase R. **(F)** Cells were treated with actinomycin D (2 μg/mL) and harvested after 0, 4, 8, 12 and 24 h. Relative expression of circLPAR3 and LPAR3 was calculated and normalized to 0 h to compare the RNA stability. **(G)** Nuclear–cytoplasmic fractionation assay was used to assess the intracellular location of circLPAR3 in drug-resistant and parent cells. U6 was considered as a nuclear control and β-actin was used as a cytoplasmic protein control. **(H)** The subcellular location of circLPAR3 was also evaluated by fluorescence *in situ* hybridization assays. Nuclei were stained with DAPI. ∗∗*P* < 0.01, ∗∗∗*P* < 0.001, and ∗∗∗∗*P* < 0.0001; ns, nonsignificant.

### circLPAR3 is regulated by METTL3-mediated m^6^A methylation

To verify the m^6^A regulation of circLPAR3, m^6^A-RIP assay results demonstrated that circLPAR3 is efficiently pulled down in the m^6^A group compared to the IgG control (Fig. 2A). m^6^A modifications are dynamically regulated by “writers” and “erasers”. To identify the specific m^6^A regulators of circLPAR3, PCR analysis revealed that METTL3, rather than other enzymes such as ALKBH5, FTO, METTL14, or WTAP, mediates changes in circLPAR3 levels upon knockdown (Fig. 2B; Fig. S2A). Potential m6A modification sites on circLPAR3 were predicted using SRAMP software,^23^ identifying six candidate sites (three high-confidence and three low-confidence) (Fig. S2B). To assess the functional relevance of these sites, mutations were introduced into the six candidate regions, and a dual-luciferase reporter assay was performed. 293T cells were co-transfected with vector/METTL3 and circLPAR3 WT/Mut constructs. The results showed that METTL3 overexpression significantly enhanced luciferase activity in the circLPAR3 WT plasmid, whereas no such effect was observed in the mutant group (Fig. 2C). Meanwhile, RIP experiments were performed again on cells with knockdown of METTL3 and control, and it was found that knockdown of METTL3 reduced the circLPAR3 abundance of the m^6^A antibody pull-down product (Fig. 2D). These results confirm that METTL3 is involved in the m^6^A modification of circLPAR3.

**Figure 2:**
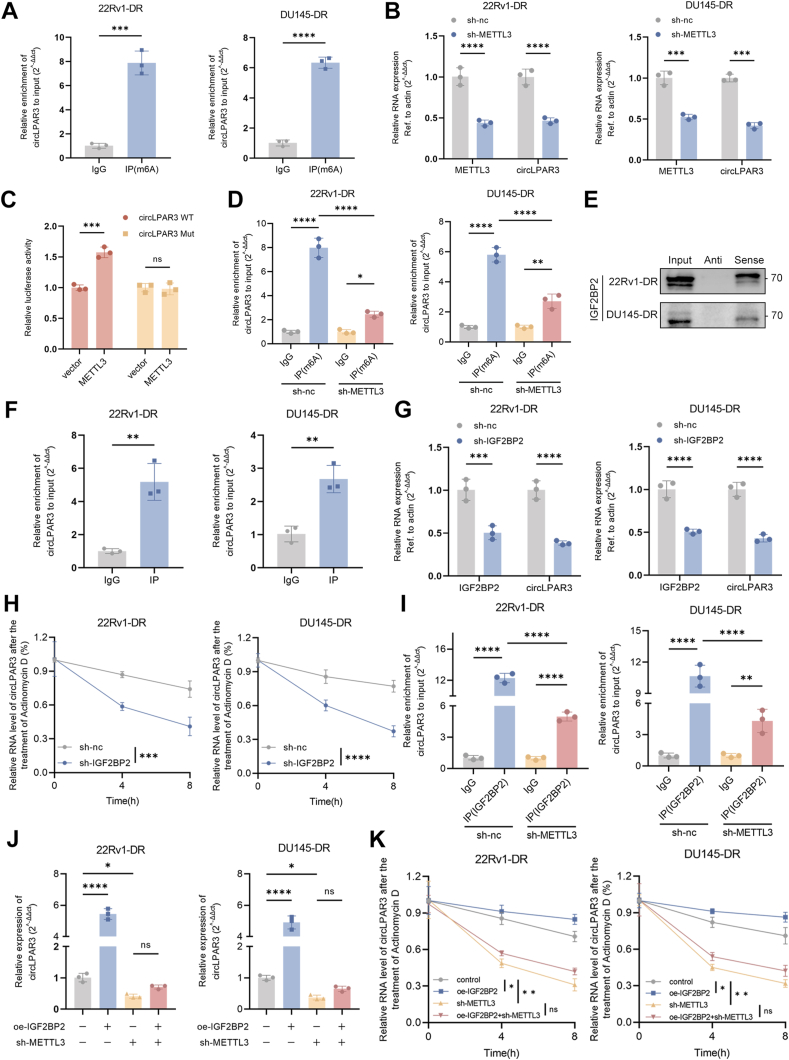
circLPAR3 was modified by m^6^A methylation via the METTL3/IGF2BP2 axis. **(A)** m^6^A-RNA immunoprecipitation assay was employed in two cell lines. Relative enrichment of m^6^A modification was calculated and normalized to input. **(B)** Expression of circLPAR3 was measured by quantitative reverse transcription PCR when METTL3 was silenced. **(C)** Wild-type or mutant plasmids of reformed luciferase reporters were transfected into 293T cells (with the overexpression of METTL3), followed by the measurement of luciferase activity. **(D)** m^6^A-RNA immunoprecipitation assay was employed in METTL3-knockdown and relative control cell lines. Relative enrichment of m^6^A modification was calculated and normalized to input. **(E)** Western blotting assays were performed to confirm that circLPAR3 could bind to IGF2BP2. **(F)** IGF2BP2-RNA immunoprecipitation assays in two cell lines were conducted to validate the binding of IGF2BP2 to circLPAR3. **(G)** Expression of circLPAR3 was detected using quantitative reverse transcription PCR when IGF2BP2 was knocked down. **(H)** The relative expression of circLPAR3 in sh-IGF2BP2-transfected cells or control was measured by quantitative reverse transcription PCR after treatment with actinomycin D. **(I)** IGF2BP2-RNA immunoprecipitation assays in METTL3-knockdown and relative control cell lines were employed to determine the binding between IGF2BP2 and circLPAR3 via m^6^A. **(J)** METTL3 was inhibited in oe-IGF2BP2 cells to determine whether increased circLPAR3 expression by IGF2BP2 was regulated by METTL3. **(K)** The relative expression of circLPAR3 in oe-IGF2BP2/sh-METTL3-transfected cells or control was measured by quantitative reverse transcription PCR after treatment with actinomycin D. ∗*P* < 0.05, ∗∗*P* < 0.01, ∗∗∗*P* < 0.001, and ∗∗∗∗*P* < 0.0001; ns, nonsignificant.

To identify the m^6^A reader of circLPAR3, a circRNA-pulldown assay followed by MS was conducted using a circLPAR3 probe. Compared to the antisense control, significant enrichment of IGF2BP2 was observed in the sense group, while IGF2BP1/3 and YTHDFs were not detected (Fig. S2C, S2D). Pull-down and Western blotting (Fig. 2E), along with RIP/qRT-PCR analyses (Fig. 2F), confirmed an interaction between circLPAR3 and IGF2BP2. Notably, knockdown of IGF2BP2 led to a reduction in circLPAR3 expression (Fig. 2G). To further investigate the role of IGF2BP2, cells with IGF2BP2 knockdown and control cells were treated with actinomycin D. The results showed that IGF2BP2 depletion accelerated circLPAR3 degradation (Fig. 2H). Additionally, RIP assays performed with IGF2BP2 antibody after METTL3 knockdown revealed a diminished enrichment of IGF2BP2 on circLPAR3 (Fig. 2I). Furthermore, METTL3 knockdown inhibited the protection of circLPAR3 expression (Fig. 2J) and stability (Fig. 2K) by IGF2BP2. These results indicate that METTL3 and IGF2BP2 collaboratively regulate circLPAR3 expression, with m^6^A modification serving as a key intermediary.

### circLPAR3 promotes docetaxel resistance in PCa cells *in vitro*

The functional role of circLPAR3 in DTX-resistant PCa cells was examined *in vitro*. Knockdown of circLPAR3 was introduced into resistant cells, while overexpression was applied to sensitive cells. The cytotoxic response to various drug concentrations was assessed using CCK-8 assays, and IC50 curves were generated. The results indicated that circLPAR3 knockdown restored sensitivity to DTX (Fig. 3A; Fig. S3A), whereas circLPAR3 overexpression diminished tumor cell sensitivity to DTX (Fig. 3B; Fig. S3B). To further evaluate cell viability, CCK-8 and EdU assays were conducted, revealing that circLPAR3 knockdown inhibited tumor cell proliferation (Fig. 3C, G; Fig. S3C and S3G), while its overexpression promoted proliferation (Fig. 3D, H; Fig. S3D and S3H). Additionally, in the presence of DTX, circLPAR3 depletion impaired colony formation (Fig. 3E; Fig. S3E), while circLPAR3 overexpression helped maintain colony formation (Fig. 3F; Fig. S3F).

**Figure 3:**
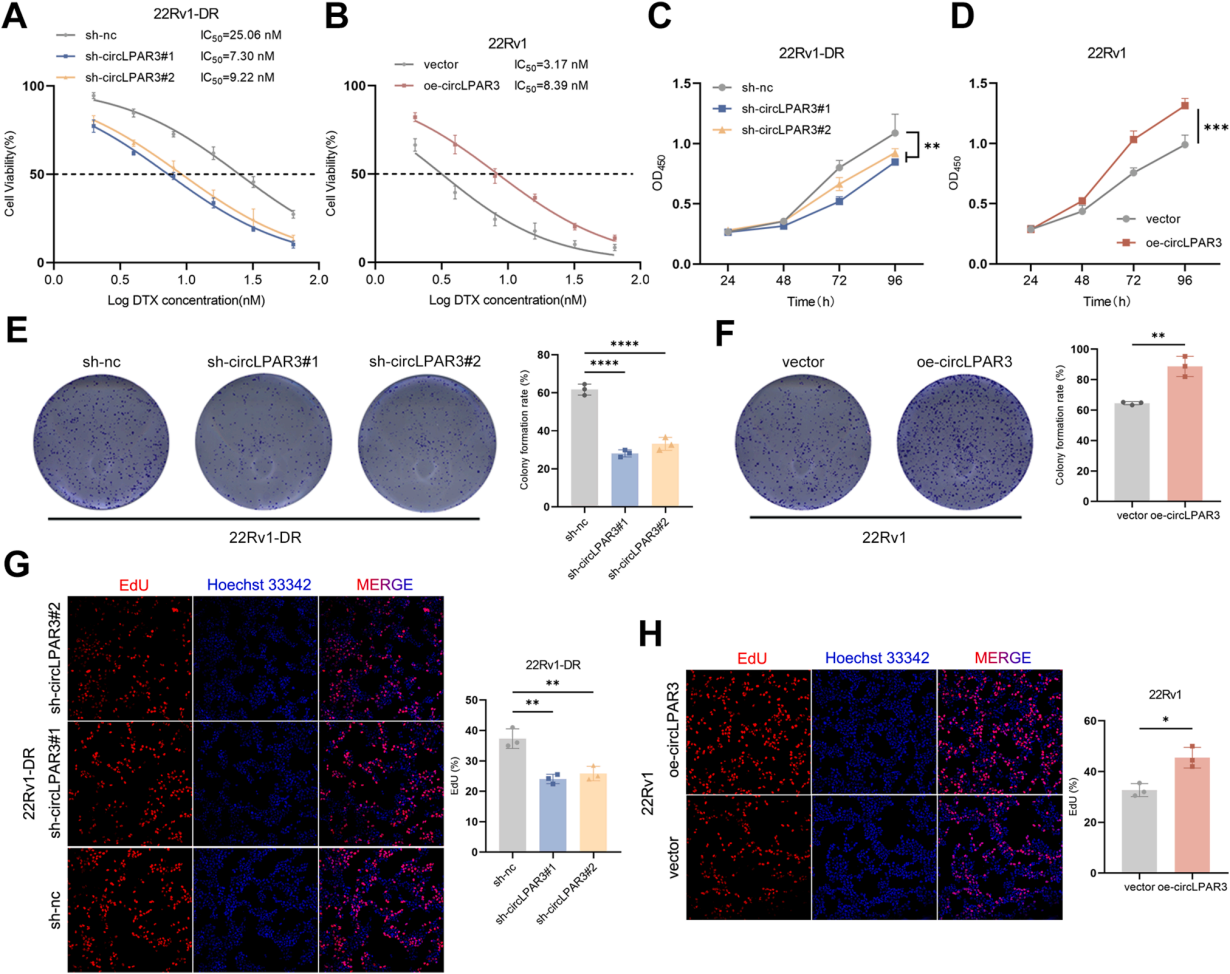
Inhibition of circLPAR3 increased docetaxel sensitivity and inhibited the proliferation of prostate cancer cells. **(A, B)** Transfected 22Rv1-DR cells (A) and 22Rv1 cells (B) were exposed to the respective indicated dose of docetaxel (2–64 μM) for 48 h and the cell viability was examined using CCK8 assays. **(C)** Knockdown of circLPAR3 in 22Rv1-DR cells and application of CCK-8 assay. **(D)** Overexpression of circLPAR3 in 22Rv1 cells and application of CCK-8 assay. **(E)** Knockdown of circLPAR3 in 22Rv1-DR cells for clone formation assay. **(F)** Overexpression of circLPAR3 in 22Rv1 cells for clone formation assay. **(G)** Knockdown of circLPAR3 in 22Rv1-DR cells for EdU assay. Scale bar, 200 mm. **(H)** Overexpression of circLPAR3 in 22Rv1 cells for EdU assay. Scale bar, 200 mm ∗*P* < 0.05, ∗∗*P* < 0.01, ∗∗∗*P* < 0.001, and ∗∗∗∗*P* < 0.0001.

### circLPAR3 inhibits ferroptosis of DTX in PCa cells

As DTX typically induces significant intracellular ROS accumulation, ROS levels in DTX-treated cells post-transfection were measured using a ROS probe. Fluorescence analysis showed that circLPAR3 knockdown led to higher ROS levels compared to DTX treatment alone (Fig. S4A), while circLPAR3 overexpression mitigated the ROS increase induced by DTX (Fig. S4B). To elucidate the mechanism via which circLPAR3 mitigates the lethal effects of DTX, several cell death inhibitors, including Ferrostatin-1, Z-VAD-FMK, and Necrostatin-1, were applied to DTX-treated cells following circLPAR3 knockdown (Fig. S4C). In both 22Rv1-DR and DU145-DR cells, knockdown of circLPAR3 resulted in a significant increase in LDH release compared to control cells, indicating enhanced cytotoxicity. However, the addition of ferroptosis inhibitors effectively reversed this toxic effect, suggesting that circLPAR3 may protect tumor cells by inhibiting ferroptosis. To further explore this, intracellular lipid peroxidation levels were assessed in DTX-treated cells using BODIPY™ 581/591 C11, a key marker of ferroptosis. Flow cytometry analysis revealed that the sh-circLPAR3+DTX group exhibited higher lipid peroxidation than the DTX-only group (Fig. 4A), while overexpression of circLPAR3 attenuated this effect (Fig. 4B). Similar results were observed for MDA and Fe^2+^ detection (Fig. 4C, D, G, H). Additionally, the sh-circLPAR3+DTX group showed increased GSH consumption compared tgo the DTX-only group (Fig. 4E), whereas circLPAR3 overexpression reduced GSH depletion (Fig. 4F). These phenotypic assays collectively demonstrate that circLPAR3 protects tumor cells by alleviating ferroptosis induced by DTX.

**Figure 4:**
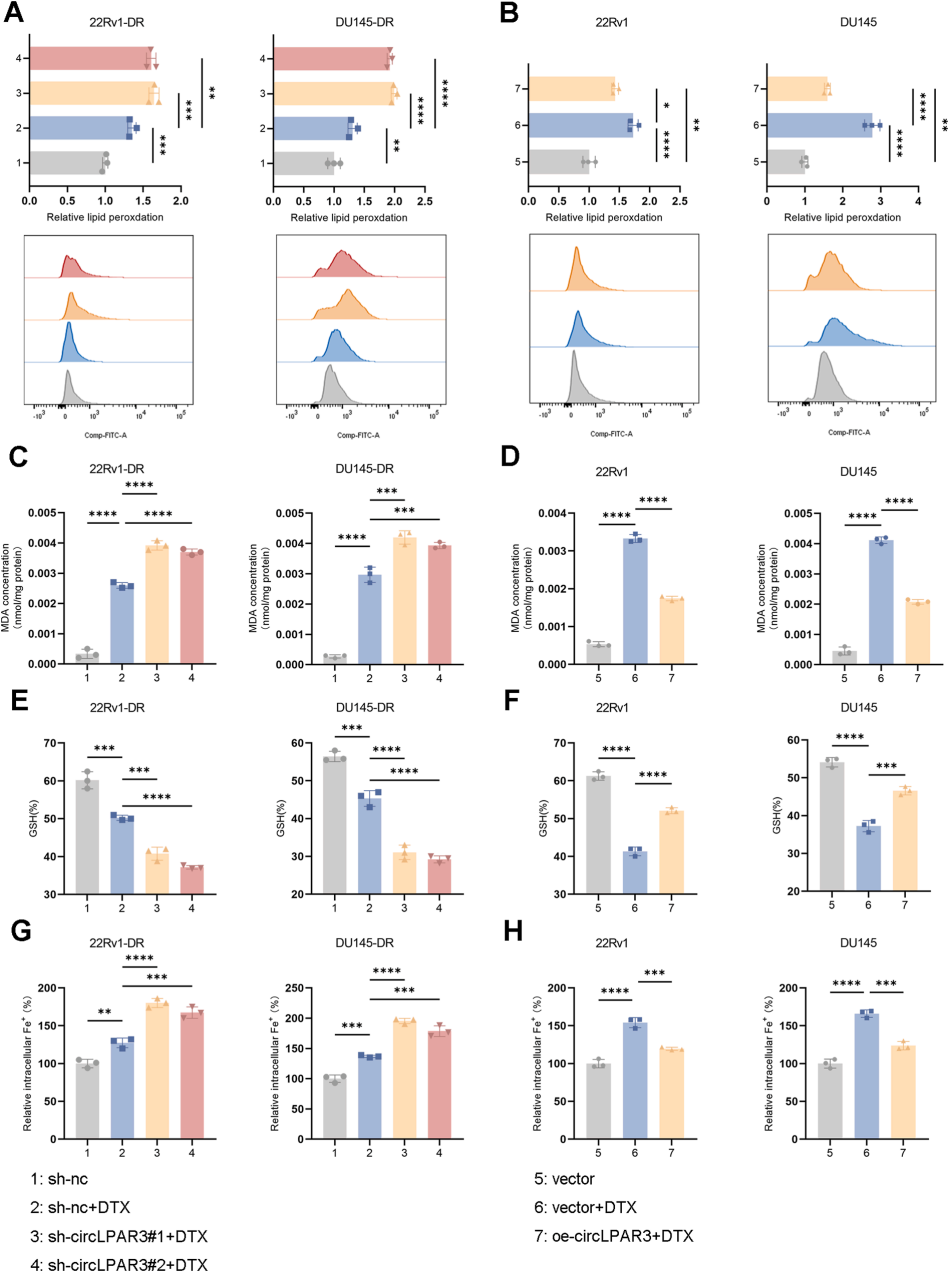
circLPAR3 protects prostate cancer cells by inhibiting docetaxel-induced ferroptosis effect. Transfected resistant and sensitive cells were exposed to the respective indicated dose of docetaxel for 48 h. **(A, B)** Flow cytometric analysis for lipid peroxidation levels using C11-BODIPY. **(C, D)** Malondialdehyde (MDA) levels were detected using MDA assay kits. **(E, F)** Concentrations of reduced glutathione (GSH) were detected using GSH assay kits. **(G, H)** Intracellular Fe^2+^ level was measured by FerroOrange using a fluorescent microplate reader. ∗*P* < 0.05, ∗∗*P* < 0.01, ∗∗∗*P* < 0.001, and ∗∗∗∗*P* < 0.0001.

### CHAC1 is a potential target gene of circLPAR3

The mechanism through which circLPAR3 inhibits ferroptosis remains unclear. Ferroptosis inducers, including erastin, RSL3, and artesunate, were applied to control and oe-circLPAR3 cells. Overexpression of circLPAR3 mitigated the cytotoxicity induced by erastin but did not affect RSL3-and artesunate-induced toxicity (Fig. S5A). RSL3 inhibits GPX4, preventing it from reducing oxidized lipids, while erastin promotes Fe^2+^ accumulation by inhibiting mitochondrial ion channels and impeding cystine uptake, thus limiting GSH synthesis. Given the distinct drug targets, it was hypothesized that circLPAR3’s protective role in ferroptosis may be linked to GSH synthesis and utilization. To explore this, researchers performed RNA-seq analysis on 22Rv1-DR cells knocked down for circLPAR3 and controls (Fig. S5B), and the differentially expressed genes were obtained and then crosslinked using the FerrDb^24^ of the ferroptosis factor set. Upset plots demonstrated the results of the crosslinking, with a total of 19 factors, including 11 drivers, 7 repressors, and 1 marker, were detected (Fig. S5C), and the heatmap demonstrated the expression of these 19 genes (Fig. S5D). Notably, the only gene among the 19 factors that was directly related to GSH metabolism was CHAC1, a molecule that acts as both a driver and a marker of ferroptosis,^25^, ^26^, ^27^, ^28^ and it was highly expressed in the sh-circLPAR3 group, which is consistent with the phenotype of enhanced iron death after knockdown of circLPAR3. Interfering with circLPAR3 expression in two drug-resistant cell lines, followed by qRT-PCR and Western blotting, revealed that downregulation of circLPAR3 led to increased CHAC1 mRNA and protein levels, and *vice versa* (Fig. S5E-S5H). These results suggest that circLPAR3 regulates CHAC1 in an inverse manner through a specific pathway.

### circLPAR3 promotes cell proliferation and ferroptosis resistance in the DTX environment by inhibiting CHAC1 expression

The IC50 of PCa against DTX was determined after further expression regulation of CHAC1 to investigate whether the protective function of circLPAR3 for cells was exerted via CHAC1. The results showed that in the resistant line, knocking down CHAC1 again on top of knocking down circLPAR3 showed a rebound of IC50 (Fig. S6A), while in the parental line, overexpressing CHAC1 again on top of overexpressing circLPAR3 showed a retraction of IC50 (Fig. S6B). This indicated that CHAC1 could rescue the upregulation of IC50 by circLPAR3 to some extent. Subsequently, the cells were grouped according to sh-nc/sh-circLPAR3/sh-circLPAR3+sh-CHAC1 in drug-resistant cells, and according to vector/oe-circLPAR3/oe-circLPAR3+oe-CHAC1 in sensitive cells, which were transfected for 48 h and then treated with DTX. The results of the CCK-8 assay and the results of clone formation assays showed that knockdown of CHAC1 restored the decreased cell proliferation caused by knockdown of circLPAR3 (Fig. S6C and S6E), and overexpression of CHAC1 similarly disrupted the proliferation-promoting effect of circLPAR3 (Fig. S6D and S6F).

Further, to investigate whether CHAC1 mediates circLPAR3’s protective effect against ferroptosis, drug-resistant cells were grouped into sh-nc/sh-circLPAR3/sh-circLPAR3+sh-CHAC1 and treated with DTX 48 h post-transfection. Lipid peroxidation in sh-CHAC1 cells was significantly reduced compared to the circLPAR3 knockdown group (Fig. 5A). In parallel, sensitive cells were grouped into vector/oe-circLPAR3/oe-circLPAR3+oe-CHAC1 and subjected to the same treatment. Overexpression of CHAC1 abolished the ferroptosis protection conferred by circLPAR3 overexpression (Fig. 5B). Corresponding results from MDA (Fig. 5C, D), GSH (Fig. 5E, F), and intracellular Fe^2+^ assays (Fig. 5G, H) confirmed the lipid peroxidation data. Collectively, these results indicate that downregulation of CHAC1 rescues ferroptosis in the absence of circLPAR3, and that circLPAR3 exerts its protective effect by inhibiting CHAC1.

**Figure 5:**
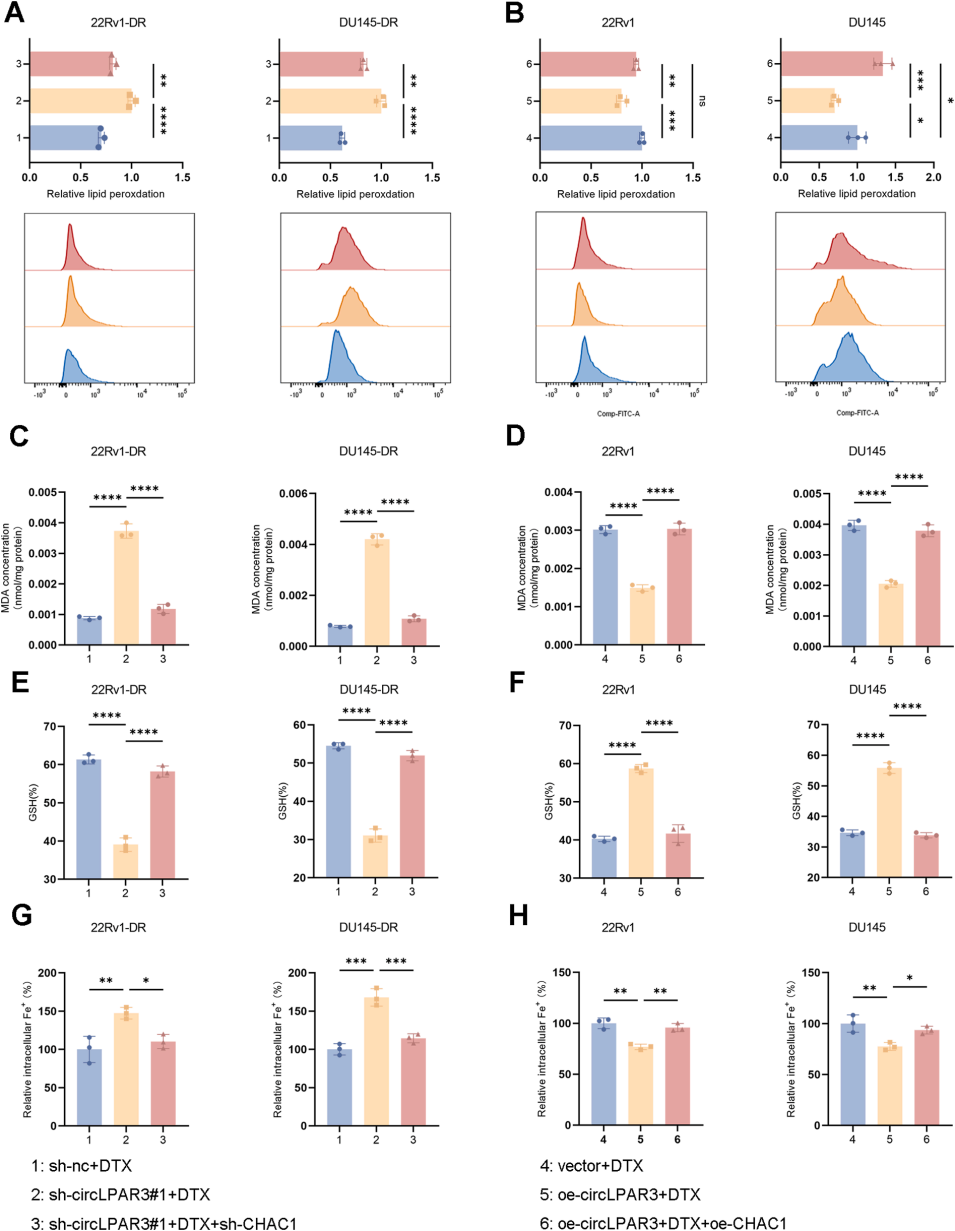
CHAC1 inhibits ferroptosis protection induced by circLPAR3. Transfected resistant and sensitive cells were exposed to the respective indicated dose of docetaxel for 48 h. **(A, B)** Flow cytometric analysis for lipid peroxidation levels using C11-BODIPY. **(C, D)** Malondialdehyde (MDA) levels were detected using MDA assay kits. **(E, F)** Concentrations of reduced glutathione (GSH) were detected using GSH assay kits. **(G, H)** Intracellular Fe^2+^ level was measured by FerroOrange using a fluorescent microplate reader. ∗*P* < 0.05, ∗∗*P* < 0.01, ∗∗∗*P* < 0.001, and ∗∗∗∗*P* < 0.0001; ns, nonsignificant.

### PCBP2 is a mediating RBP for circLPAR3 and CHAC1

Given the cytoplasmic localization of circLPAR3, it is hypothesized that circLPAR3 regulates CHAC1 at the post-transcriptional rather than transcriptional level. Unlike the typical positive regulation of target genes through the ceRNA mechanism, circLPAR3’s regulation of CHAC1 is inversely correlated. Therefore, it was speculated that circLPAR3 may regulate CHAC1 by interacting with a specific RBP. Integrating circRNA-pulldown/MS data with cross-linking and immunoprecipitation (CLIP)-sequencing data from ENCORI,^29^ two common RBPs, PCBP2 and IGF2BP2, were identified (Fig. S7A). Further analysis using the catRAPID database^30^ to simulate binding interactions between the protein sequences of these RBPs and the CHAC1 transcript revealed that PCBP2 ranked higher (Fig. S7B). MS data from circLPAR3 probe pull-downs also detected three highly abundant peptides corresponding to PCBP2 (Fig. S2C). These results suggest that PCBP2 is more likely involved in mediating circLPAR3’s regulation of CHAC1 expression. circRNA pull-down/Western blotting (Fig. 6A) and RIP/qRT-PCR assays (Fig. 6B) confirmed an interaction between circLPAR3 and PCBP2, while FISH-IF analysis revealed co-localization of both in the cytoplasm (Fig. 6C). In addition, circLPAR3 and PCBP2 were knocked down separately, and the altered expression levels of each other were detected using qRT-PCR and Western blotting, and it was found that the knockdown of circLPAR3 had no effect on PCBP2 expression (Fig. S7C, S7D), and there was no expression regulation of circLPAR3 by PCBP2 (Fig. S7E).

**Figure 6:**
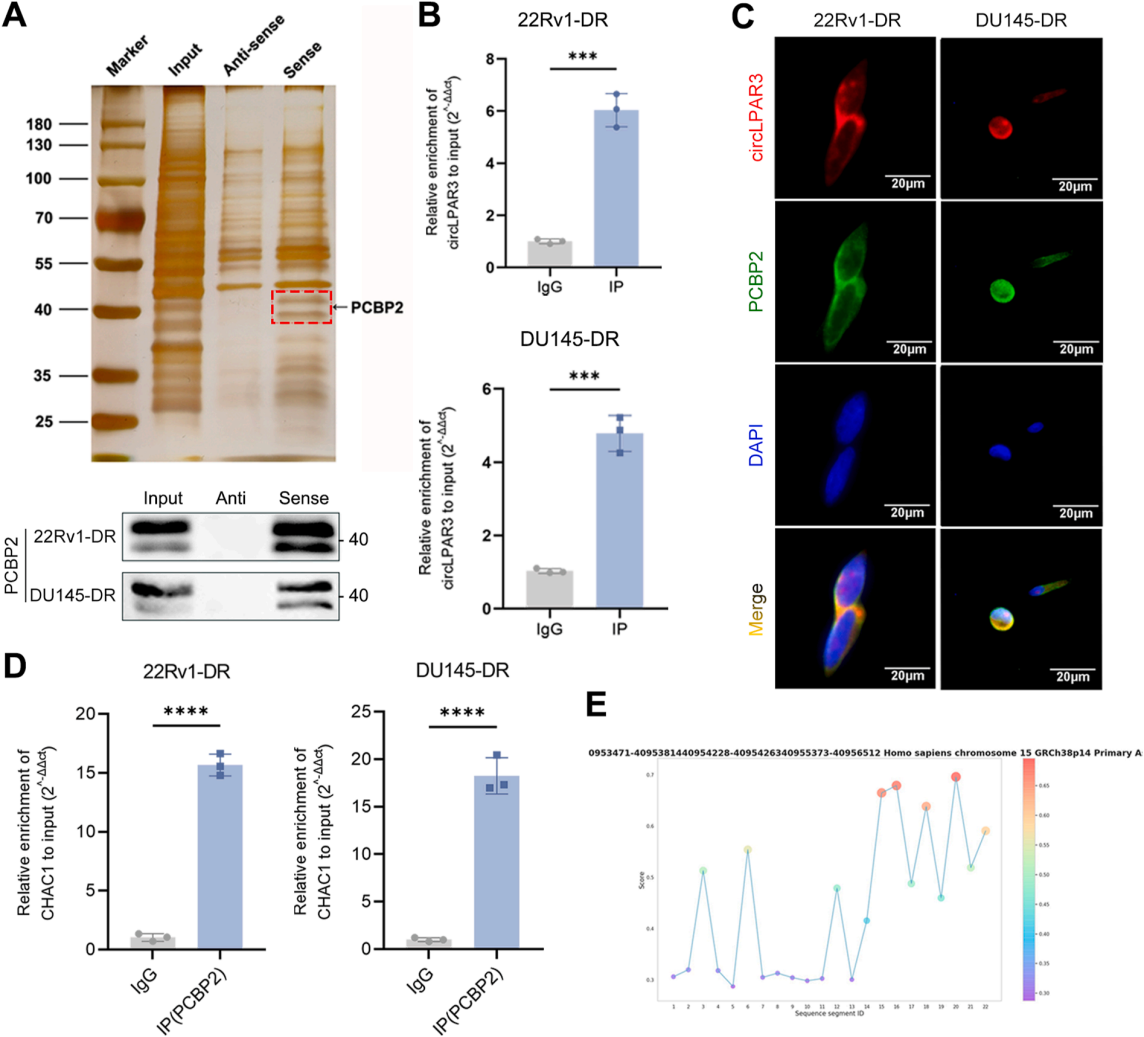
PCBP2 is a mediating RBP for circLPAR3 and CHAC1. **(A)** Silver staining assay and Western blotting assay were conducted to verify the binding of circLPAR3 to PCBP2. **(B)** PCBP2-RNA immunoprecipitation assay was conducted to verify the binding of PCBP2 to circLPAR3. **(C)** Fluorescence *in situ* hybridization and immunofluorescence assays were carried out to indicate the co-localization of circLPAR3 and PCBP2. **(D)** PCBP2-RNA immunoprecipitation assay was conducted to verify the binding of PCBP2 to CHAC1. **(E)** Binding of PCBP2 on CHAC1 mRNA was predicted using RBPsuite Predicted. ∗∗∗*P* < 0.001 and ∗∗∗∗*P* < 0.0001.

Similarly, the pull-down of PCBP2 on CHAC1 was verified by RIP experiments (Fig. 6D), confirming that PCBP2 is the RBP of CHAC1. The binding of PCBP2 protein to CHAC1 mRNA was predicted using the RBPsuite database, and it was found that the 3′ end close to the mRNA showed a relatively high binding score (Fig. 6E).

### circLPAR3 interacts with PCBP2 to weaken the stability of CHAC1 mRNA

PCBP2 is an RBP with a high affinity for poly(C) and contains three hnRNP K homology (KH) domains, a hallmark of its family. Known for its diverse roles in post-transcriptional regulation, PCBP2 has been implicated in mRNA pre-splicing, stabilization, and translation control.^31^ Specifically, PCBP2 stabilizes mRNAs such as alpha-globin and neurofilament mRNAs by binding to their 3′UTR. Upon knockdown or overexpression of PCBP2 in two cell lines, both RNA and protein levels of CHAC1 were found to fluctuate in parallel with changes in PCBP2 expression (Fig. S7F-S7I).

**Figure 7:**
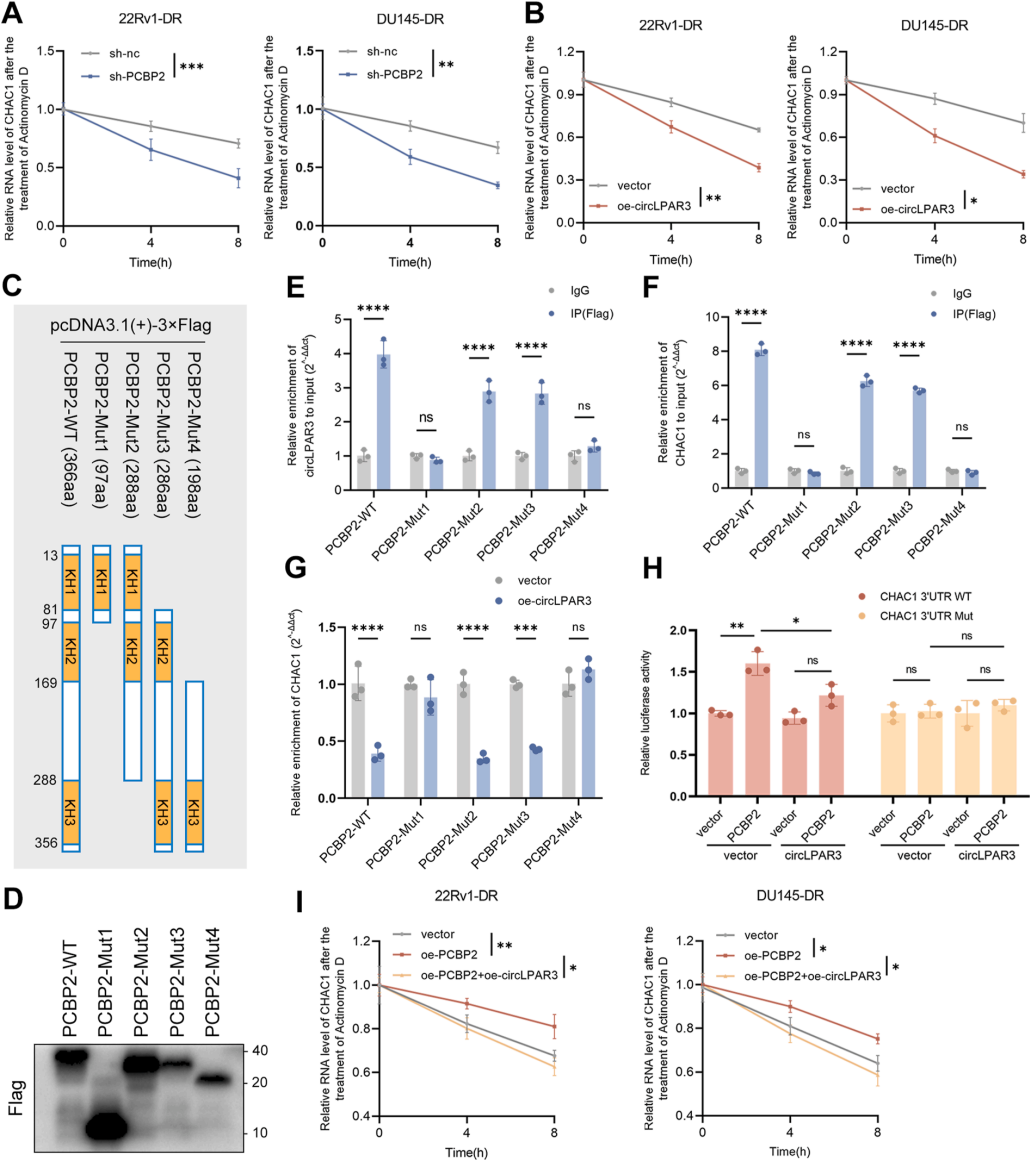
circLPAR3 inhibits CHAC1 stability by weakening the binding of IGF2BP2 to CHAC1. **(A)** The remaining CHAC1 mRNA levels in sh-PCBP2-transfected or control cells treated with actinomycin D at different time points were determined by quantitative reverse transcription PCR. **(B)** The remaining CHAC1 mRNA levels in oe-circLPAR3-transfected or control cells treated with actinomycin D at different time points were determined by quantitative reverse transcription PCR. **(C)** Schematic diagram of PCBP2 structural domain mutant plasmid. **(D)** Western blotting was conducted to verify the transfection of mutant plasmids. **(E)** Flag-RNA immunoprecipitation assay was carried out to determine the region of IGF2BP2 that could bind to circLPAR3. **(F)** Flag-RNA immunoprecipitation assay was carried out to determine the region of IGF2BP2 that could bind to CHAC1. **(G)** Flag-RNA immunoprecipitation assay was performed to detect the abundance of CHAC1 precipitated with overexpression of circLPAR3 or control. **(H)** Relative luciferase activity of CHAC1 3′ UTR-WT or CHAC1 3′ UTR-MT that was detected in PCBP2 overexpression or relative control 293T cells with or without overexpression of circLPAR3. **(I)** Actinomycin D treatment assay showed that overexpression of IGF2BP1 in cells disrupted the maintenance of CHAC1 mRNA stability by PCBP2. ∗*P* < 0.05, ∗∗*P* < 0.01, ∗∗∗*P* < 0.001, and ∗∗∗∗*P* < 0.0001; ns, nonsignificant.

To verify whether the altered expression of CHAC1 was mediated by altered stability, actinomycin D was used to treat knockdown PCBP2 and control cells, and qRT-PCR was used to detect the relative expression changes of CHAC1 between different time points. It was found that mRNA degradation of CHAC1 was faster after PCBP2 knockdown (Fig. 7A), and the same result was achieved by overexpressing circLPAR3 (Fig. 7B). Due to the centrality of the KH structural domain in the function of PCBP2 as an RBP, we constructed a truncated body-tagged plasmid with mutations in the KH structural domain (Fig. 7C, D) and performed RIP experiments after transfection with the aim of exploring the binding mode of PCBP2 to circLPAR3 and CHAC1. The results showed that the tagged proteins were able to pull down a high abundance of circLPAR3 (Fig. 7E) and CHAC1 (Fig. 7F) in the groups of WT, Mut2, and Mut3, suggesting that PCBP2 binds to these two proteins through its KH2 structural domain. This has led to speculation as to whether a competitive binding mechanism intervenes in the inhibition of CHAC1 by circLPAR3. The truncated somatic plasmid was further transfected into cells overexpressing circLPAR3, and RIP experiments were performed to observe the abundance of CHAC1 in the down-regulation. The results showed that overexpression of circLPAR3 inhibited the pull-down of CHAC1 by tagged proteins in the groups of WT, Mut2 and Mu3 (Fig. 7G). Additionally, a recombinant luciferase reporter plasmid with a mutated 3′UTR sequence was constructed. PCBP2 increased luciferase activity in the wild-type plasmid, but no change was observed in the mutant plasmid (Fig. 7H). These results suggest that PCBP2 binds to the 3′UTR of CHAC1 mRNA, stabilizing its expression. Moreover, overexpression of circLPAR3 inhibited the increase in luciferase activity induced by PCBP2 in the wild-type group (Fig. 7H). Finally, the results of the RNA pull-down assay showed that overexpression of circLPAR3 inhibited the protection of PCBP2 against CHAC1 mRNA stability (Fig. 7I). Thus, circLPAR3 achieves a reverse regulatory effect on CHAC1 by competitively binding to PCBP2 and weakening the stabilizing effect of PCBP2 on CHAC1 mRNA.

### circLPAR3 promotes docetaxel resistance in PCa *in vivo*

**Figure 8:**
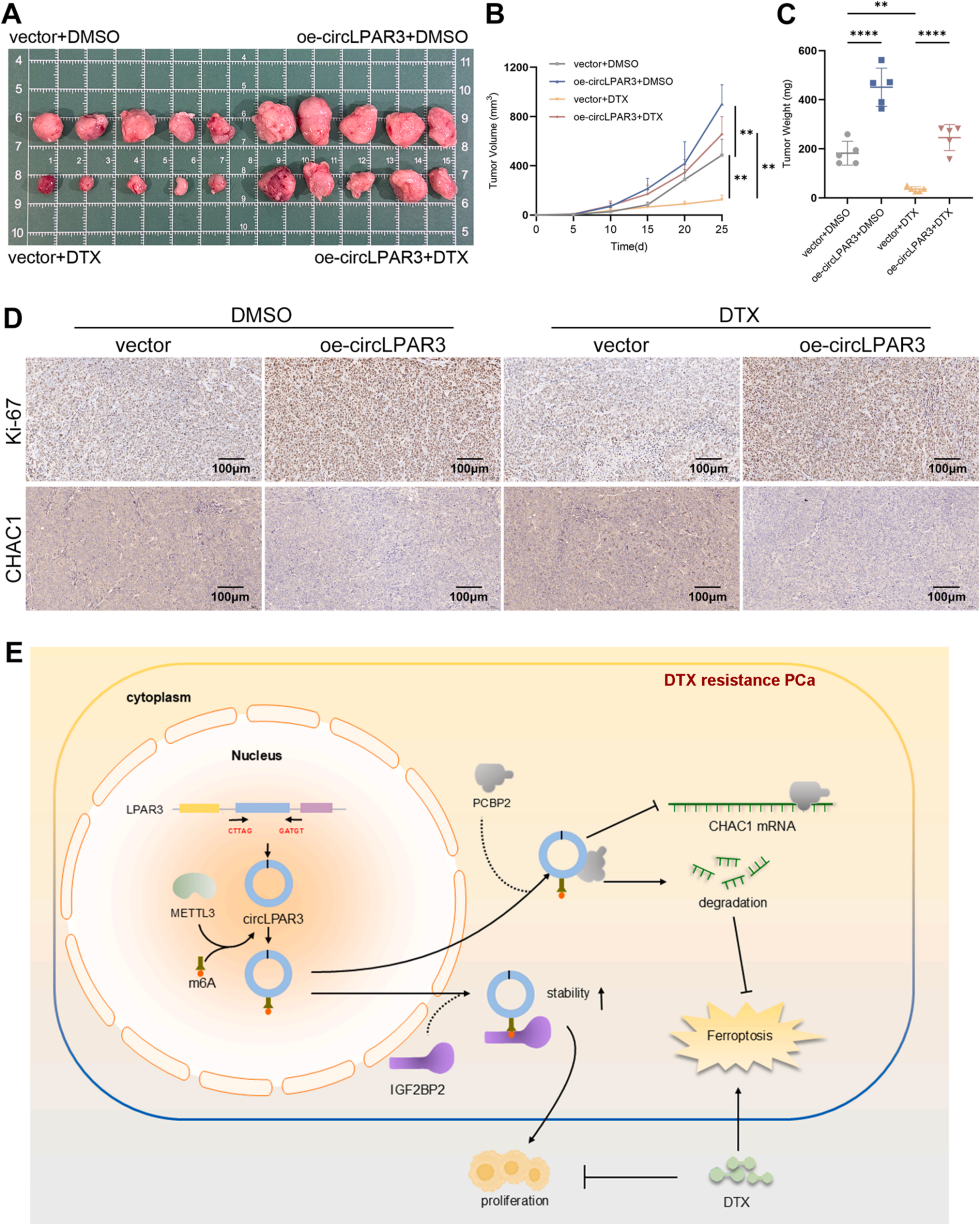
circLPAR3 increased prostate cancer cell proliferation and docetaxel resistance in CDX models. **(A)** Representative images of xenograft tumors in the oe-circLPAR3 22Rv1-DR group and its control group (vector) after treatment with docetaxel or DMSO. **(B, C)** Tumor growth curves were plotted and tumor weights were measured. **(D)** Immunohistochemistry assessment of Ki-67 and CHAC1 expression in the oe-circLPAR3 group and its control group. **(E)** METTL3/IGF2BP2-regulated circLPAR3 promotes proliferation of drug-resistant cells and confers ferroptosis resistance to docetaxel-resistant prostate cancer cells by binding to PCBP2 and inhibiting the latter’s stabilizing effect on CHAC1 mRNA. ∗∗*P* < 0.01 and ∗∗∗∗*P* < 0.0001.

To assess the functional role of circLPAR3 in DTX-resistant PCa cells *in vivo*, 22Rv1-DR cells stably transfected with sh-circLPAR3 or control vector were subcutaneously injected into BALB/c nude mice. After tumor formation, intraperitoneal injections of DMSO or DTX were administered (Fig. 8A). The data showed that circLPAR3 overexpression significantly increased tumor volume and weight, while also reducing DTX sensitivity in 22Rv1-DR cells (Fig. 8B, C). Immunohistochemistry analysis revealed elevated Ki-67 expression and decreased CHAC1 expression in the oe-circLPAR3 group compared to the control (Fig. 8D). These results indicate that circLPAR3 not only promotes tumor growth but also plays a critical role in modulating the sensitivity of PCa cells to DTX chemotherapy.

## Discussion

Evidence from multiple clinical studies has established DTX as a first-line treatment for mCRPC. However, most patients develop DTX resistance after an initial period of efficacy, posing challenges for disease control and quality of life.^32^^,^^33^ In this study, we reveal that m6A-modified circLPAR3 regulates PCa sensitivity to DTX via the PCBP2/CHAC1 pathway.

circLPAR3 has been rarely reported in tumors, yet existing studies highlight its potential in cancer therapy. It has been linked to suppressed radiotherapy sensitivity in PCa^34^ and, in ovarian cancer, enhances cisplatin resistance via a ceRNA mechanism.^35^ Our sequencing data and functional validation demonstrate that circLPAR3 is highly expressed in multiple drug-resistant cell lines, and its overexpression correlates with DTX chemoresistance in PCa cells, suggesting its potential as a therapeutic target.

Furthermore, our study shows that circLPAR3 is regulated by METTL3/IGF2BP2-mediated m6A modification, enhancing its stability. Notably, Xu and Niu reported elevated IGF2BP2 expression in both DTX-resistant PCa cells and DTX-treated patients,^7^ suggesting that IGF2BP2 recognition and stabilization contribute to circLPAR3 upregulation in resistant cells. This epigenetic crosstalk provides new research directions. First, given circLPAR3’s greater stability compared to its linear counterpart LPAR3 and m6A-mediated resistance to degradation, it could serve as a potential biomarker for *in vitro* diagnosis and chemotherapy efficacy.^36^ Second, CWI1-2,^37^ an IGF2BP2-targeted inhibitor, remains unexplored in PCa, warranting investigation into its role and mechanisms in DTX-resistant PCa.

Growing evidence suggests that DTX efficacy can be enhanced by ferroptosis sensitization.^16^^,^^17^^,^^38^ Our preliminary data indicate that DTX induces lipid peroxidation in PCa cells. Here, using various non-programmed cell death inhibitors alongside DTX in circLPAR3-knockdown resistant cells, along with LDH cytotoxicity assays, we found that ferroptosis inhibition produced the most statistically significant effects. Functionally, circLPAR3 expression changes alter ferroptotic phenotypes in PCa cells under DTX treatment, strengthening the link between DTX and ferroptosis. Combining ferroptosis inducers targeting protective mechanisms may thus improve DTX efficacy.

CHAC1, an emerging ferroptosis regulator, promotes ferroptosis by degrading GSH and disrupting redox balance.^26^^,^^28^ Our findings show that CHAC1, a circLPAR3 target gene, is suppressed in resistant cells, potentially boosting antioxidant capacity and conferring ferroptosis resistance and DTX tolerance. Through circRNA pull-down, mass spectrometry, and CLIP-seq, we identified PCBP2 as the intermediary RBP by which circLPAR3 inhibits CHAC1. PCBP2 stabilizes CHAC1 mRNA by binding its 3′UTR, while circLPAR3 competitively binds to PCBP2’s KH2 domain to disrupt this interaction. Intriguingly, PCBP2 not only stabilizes mRNA but also enhances translation and regulates transcription. It also functions as an iron chaperone in ferroptosis^39^^,^^40^ and can also interact with SLC7A11 and GPX4.^41^^,^^42^ Thus, whether circLPAR3-PCBP2 influences ferroptosis via other targets merits further exploration, possibly through metabolomic or proteomic analyses.

In summary, we identify circLPAR3 as a novel DTX resistance-associated circRNA in PCa. Upregulated in resistant cells, it promotes proliferation and suppresses ferroptosis. Mechanistically, circLPAR3 is m6A-modified by METTL3 and stabilized by IGF2BP2, which is overexpressed in resistant cells. Cytoplasmic circLPAR3 competitively binds to PCBP2, impairing its stabilization of CHAC1 mRNA (as illustrated in Figure 8E).

## CRediT authorship contribution statement

**Yetao Zhang:** Conceptualization, Methodology, Software, Data curation, Investigation, Project administration, Supervision, Visualization, Formal analysis, Resources, Validation, Writing – review & editing, Funding acquisition, Writing – original draft. **Yuxiang Dong:** Validation, Data curation, Investigation, Software, Formal analysis. **Kai Li:** Validation, Investigation, Formal analysis, Methodology, Data curation, Visualization, Software. **Tong Zhao:** Software, Investigation, Writing – review & editing, Data curation, Validation. **Yongshan Li:** Validation, Visualization, Investigation, Methodology. **Mingyang Pang:** Methodology, Validation, Investigation, Visualization. **Yong Wei:** Investigation, Validation, Supervision, Writing – review & editing, Methodology, Visualization. **Bing Yao:** Data curation, Conceptualization, Resources, Project administration, Writing – review & editing, Formal analysis, Validation. **Qingyi Zhu:** Formal analysis, Project administration, Supervision, Funding acquisition, Resources, Validation, Conceptualization, Writing – original draft, Data curation, Investigation, Software, Writing – review & editing.

## Ethics declaration

The animal research protocol was approved by the Animal Care and Use Committee of the Laboratory Animal Center of Nanjing Medical University. The study adhered to the guidelines established by the committee.

## Data availability

The datasets that support the conclusions of this article are included in the article and its accompanying documentation. Additional support can be obtained by contacting the corresponding authors.

## Conflict of interests

The authors declared no conflict of interests.

## Figures and Tables

**figs1:**
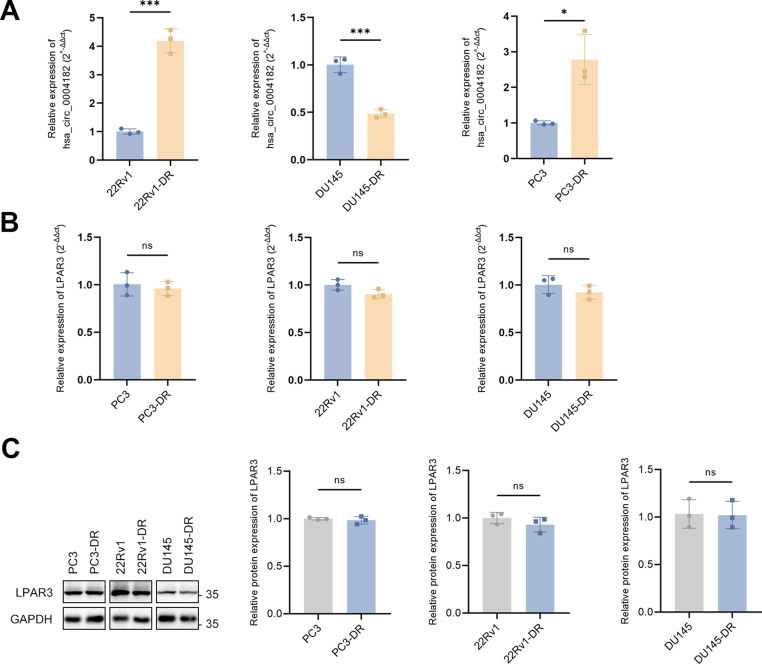


**figs2:**
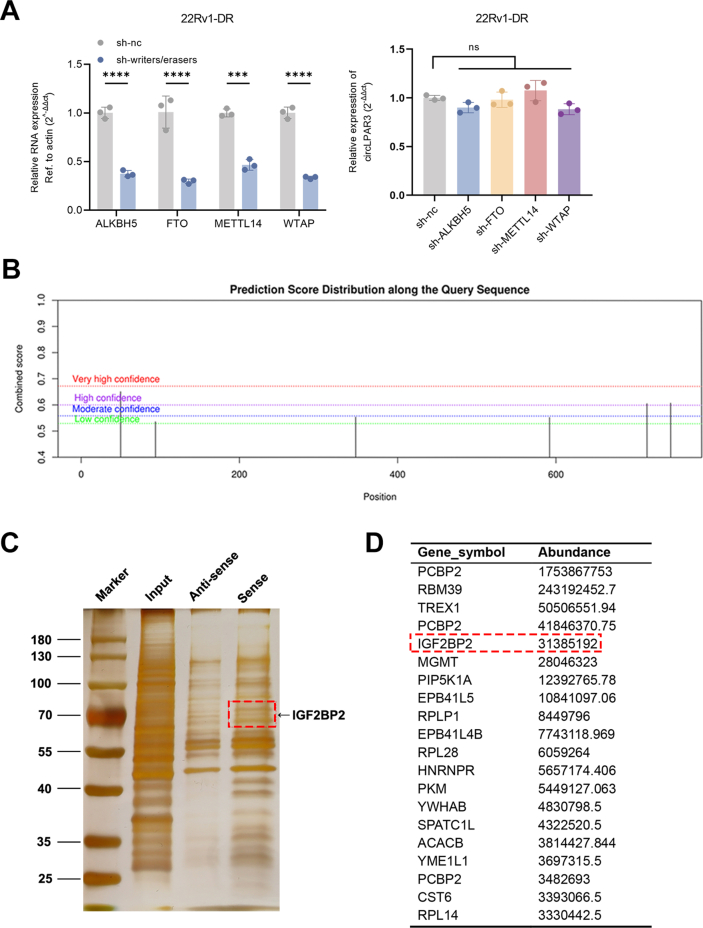


**figs3:**
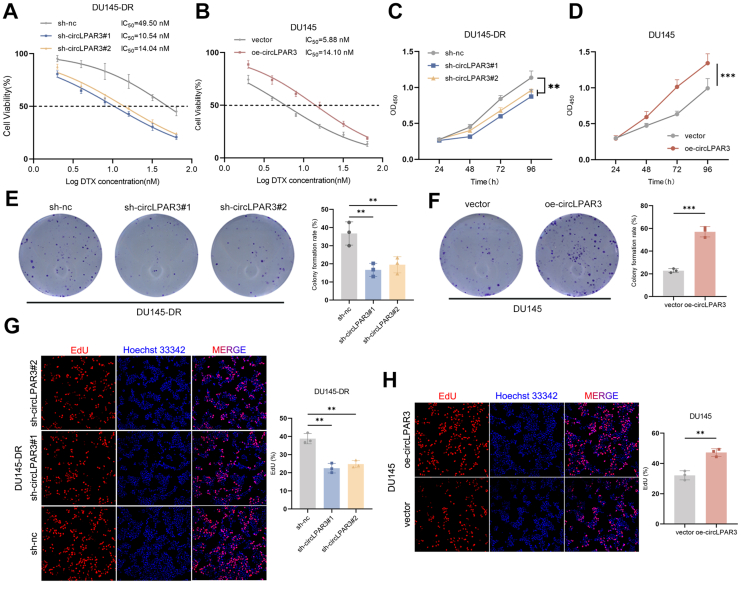


**figs4:**
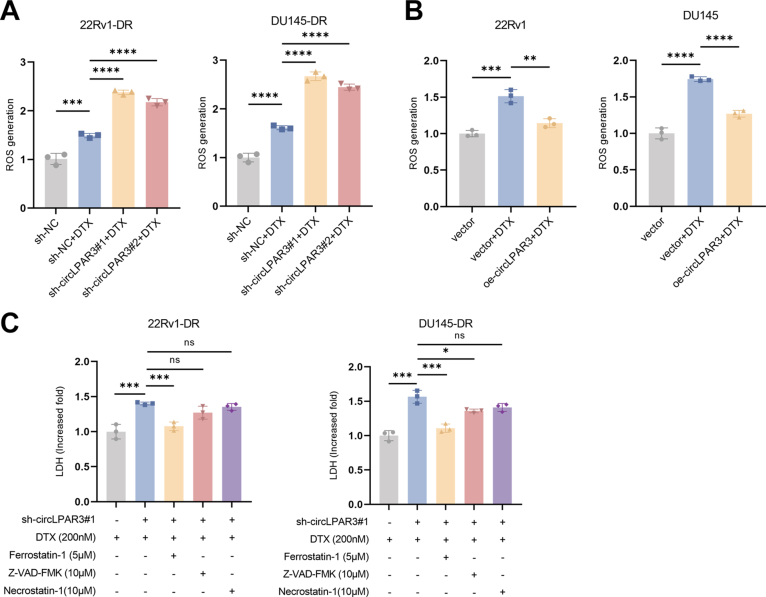


**figs5:**
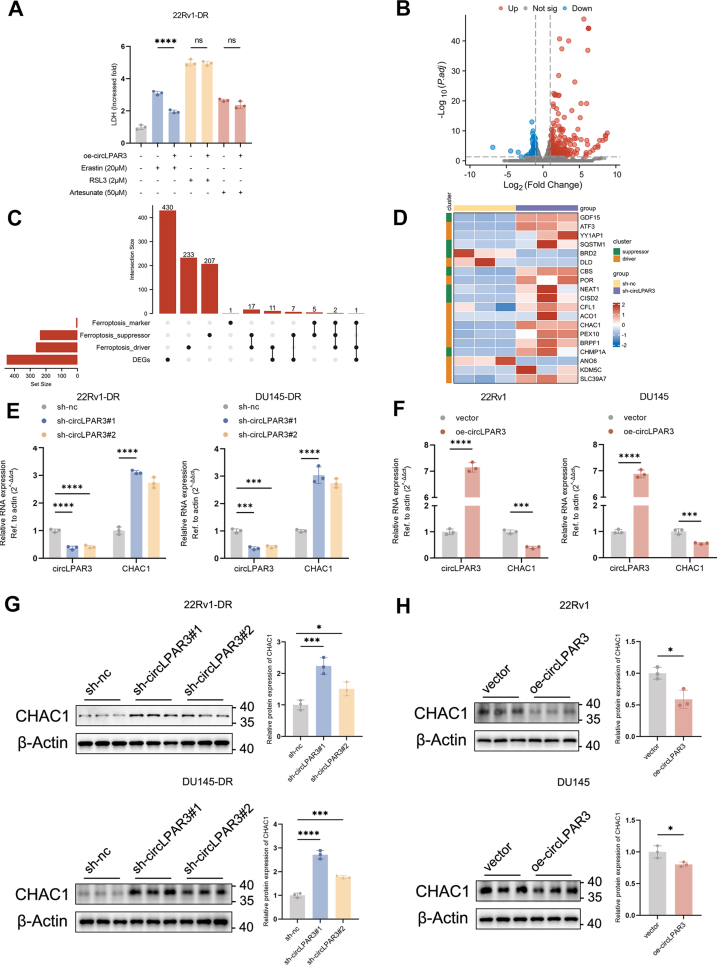


**figs6:**
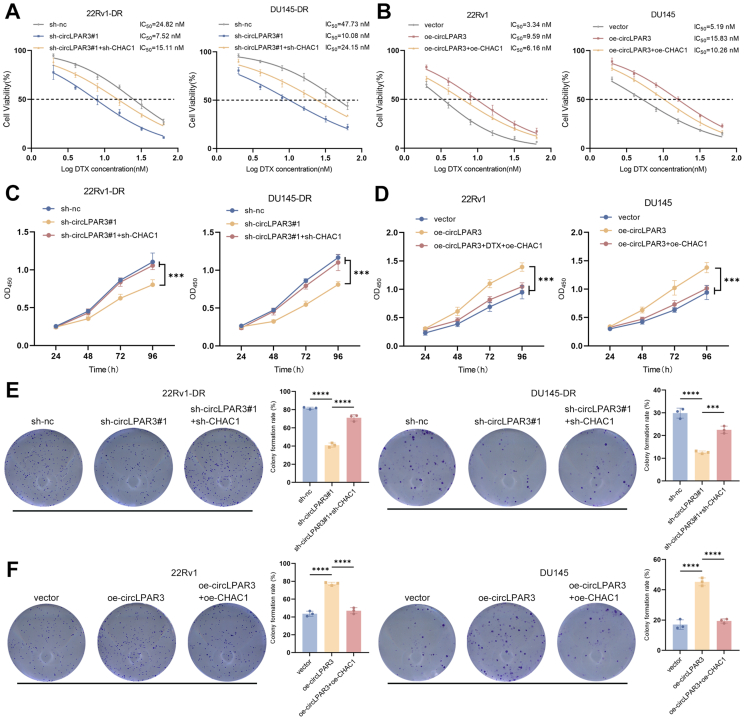


**figs7:**