# Supplementary material for: N6-methyladenosine-modified circLPAR3 drives docetaxel resistance in prostate cancer by suppressing ferroptosis through the PCBP2/CHAC1 axis
Source: Genes Dis. 2025 Aug 22;13(6):101816. doi: 10.1016/j.gendis.2025.101816 (PMC13487614; doi:10.1016/j.gendis.2025.101816)
Supplement: Supplementary file 1 [file mmc1.docx]

**Supplementary Figure 1**

**A,** The expression of hsa_circ_0004182 in docetaxel-resistant prostate cancer cells (PC3-DR, 22Rv1-DR and DU145-DR) and sensitive cell lines (PC3, 22Rv1 and DU145). **B-C,** The expression of LPAR3 in docetaxel-resistant prostate cancer cells (PC3-DR, 22Rv1-DR and DU145-DR) and sensitive cell lines (PC3, 22Rv1 and DU145). The expression of hsa_circ_0004182 in docetaxel-resistant prostate cancer cells (PC3-DR, 22Rv1-DR and DU145-DR) and sensitive cell lines (PC3, 22Rv1 and DU145). *, P < 0.05; ***, P < 0.001; ns, nonsignificant.

**Supplementary Figure 2**

**A,** Knockdown efficiency of several writers and erasers, and expression of circLPAR3. **B,** Potential m^6^A sites of circLPAR3 were predicted via the online tool SRAMP. **C,** RNA–protein pulldown experiment and silver staining assay were conducted to investigate the possible proteins, which could bind circLPAR3. **D,** Top 20 for mass spectrometry of RNA-pulldown samples. ***, P < 0.001; ****, P < 0.0001; ns, nonsignificant.

**Supplementary Figure 3**

Transfected DU145-DR cells **(A)** and DU145 cells **(B)** were exposed to the respective indicated dose of docetaxel (2–64 μM) for 48 h and the cell viability was examined using CCK8 assays. **C,** Knockdown of circLPAR3 in DU145-DR cells and application of CCK-8 assay. **D,** Overexpression of circLPAR3 in DU145 cells and application of CCK-8 assay. **E,** Knockdown of circLPAR3 in DU145-DR cells and clone formation assay. **F,** Overexpression of circLPAR3 in DU145 cells for clone formation assay. **G,** Knockdown of circLPAR3 in DU145-DR cells for EdU assay. Scale bar is 200 mm. **H,** Overexpression of circLPAR3 in DU145 cells for EdU assay. Scale bar is 200 mm. *, P < 0.05; **, P < 0.01; ***, P < 0.001.

**Supplementary Figure 4**

**A-B,** Fluorescence counting and quantitative analysis of intracellular ROS in resistant and sensitive cells stained with DCFH-DA. **C,** Resistant cells transfected with sh-circLPAR3 were exposed to docetaxel for 48 hours after treatment with the indicated concentrations of cell death inhibitors and cytotoxicity was measured by LDH. *, P < 0.05; **, P < 0.01; ***, P < 0.001; ****, P < 0.0001; ns, nonsignificant.

**Supplementary Figure 5**

**A,** 22Rv1-DR cell transduced with oe-circLPAR3 were treated with indicated cell death inducers and concentrations for 24 hours, cytotoxicity was detected by LDH assay. **B,** Volcano plot showing mRNA sequencing of differentially expressed genes in knockdown circLPAR3 and control. **C,** Upset plot showing the intersection of differentially expressed genes with ferroptosis-associated factors. **D,** Heatmap showing 19 ferroptosis-related differential genes. QRT-PCR **(E-F)** and western blot **(G-H)** assays were performed to validate the CHAC1 expression after knockdown or overexpression of circLPAR3. *, P < 0.05; ***, P < 0.001; ****, P < 0.0001; ns, nonsignificant.

**Supplementary Figure 6**

Transfected 22Rv1-DR cells **(A)** and 22Rv1 cells **(B)** were exposed to the respective indicated dose of docetaxel (2–64 μM) for 48 h and the cell viability was examined using CCK8 assays. CHAC1 was knocked down in sh-circLPAR3-resistant cells with CCK-8 assay **(C)** and clone formation assay **(E)** to determine whether decreasing CHAC1 rescued the lack of proliferation inhibition of circLPAR3 in resistant cells. CHAC1 was overexpressed in oe-circLPAR3-sensitive cells and assayed with CCK-8 **(D)** and clone formation assay **(F)** to determine whether increasing CHAC1 could hinder the proliferation promotion of circLPAR3 on sensitive cells. Cells of all groups were exposed to DTX. ***, P < 0.001; ****, P < 0.0001.

**Supplementary Figure 7**

**A,** The results of ENCORI database predictions and mass spectrometry analysis were intersected, and proteins binding to both circLPAR3 and CHAC1 was found. **B,** The binding grade of CHAC1 to PCBP2 was assessed using the catPAPID algorithm. **C-E,** Effects of interference with circLPAR3 and PCBP2 on their expression detected by qRT-PCR and western blot. **F-I,** QRT-PCR and western blot assays were performed to validate the CHAC1 expression after overexpression of PCBP2. *, P < 0.05; **, P < 0.01; ***, P < 0.001; ****, P < 0.0001; ns, nonsignificant.
