# Supplementary material for: N6-methyladenosine-modified circLPAR3 drives docetaxel resistance in prostate cancer by suppressing ferroptosis through the PCBP2/CHAC1 axis
Source: Genes Dis. 2025 Aug 22;13(6):101816. doi: 10.1016/j.gendis.2025.101816 (PMC13487614; doi:10.1016/j.gendis.2025.101816)
Supplement: Supplementary file 2 [file mmc2.docx]

**Table S1. The sequences of primers used for qRT-PCR.**

| Primers | Sequence (5’-3’) |
| --- | --- |
| circLPAR3 (forward) | GTGGTGTACCTGCGGATCTA |
| circLPAR3 (reverse) | TGGAHAAHTGAACATCCTAAGACA |
| IGF2BP2 (forward) | AGCTAAGCGGGCATCAGTTTG |
| IGF2BP2 (reverse) | CCGCAGCGGGAAATCAATCT |
| PCBP2 (forward) | AGGCAGGTTACCATCACTGG |
| PCBP2 (reverse) | CATTGTTCTAGCTGCTCCCC |
| CHAC1 (forward) | GACGCTCCTTGAAGATCATGAG |
| CHAC1 (reverse) | CAGCAAGTATTCAAGGTTGTGG |
| LPAR3 (forward) | GGGTCCAGCATACCACAAAC |
| LPAR3 (reverse) | CAACGTCTTGTCTCCGCATA |
| METTL3 (forward) | AGATGGGGTAGAAAGCCTCCT |
| METTL3 (reverse) | TGGTCAGCATAGGTTACAAGAGT |
| METTL14 (forward) | GTTGGAACATGGATAGCCGC |
| METTL14 (reverse) | CAATGCTGTCGGCACTTTCA |
| WTAP (forward) | CTTCCCAAGAAGGTTCGATTGA |
| WTAP (reverse) | TCAGACTCTCTTAGGCCAGTTAC |
| FTO (forward) | ACTTGGCTCCCTTATCTGACC |
| FTO (reverse) | TGTGCAGTGTGAGAAAGGCTT |
| ALKBH5 (forward) | CGGCGAAGGCTACACTTACG |
| ALKBH5 (reverse) | CCACCAGCTTTTGGATCACCA |
| U6 (forward) | CTCGCTTCGGCAGCACA |
| U6 (reverse) | AACGCTTCACGAATTTGCGT |
| Actin (forward) | AGTTGCGTTACACCCTTTCTTG |
| Actin (reverse) | GCTGTCACCTTCACCGTTCC |
