# Supplementary material for: N6-methyladenosine-modified circLPAR3 drives docetaxel resistance in prostate cancer by suppressing ferroptosis through the PCBP2/CHAC1 axis
Source: Genes Dis. 2025 Aug 22;13(6):101816. doi: 10.1016/j.gendis.2025.101816 (PMC13487614; doi:10.1016/j.gendis.2025.101816)
Supplement: Supplementary file 3 [file mmc3.docx]

**Table S2. The sequences of shRNAs used in this project.**

| siRNA/shRNA | Sequence (5’-3’) |
| --- | --- |
| circLPAR3 shRNA-1 | GUGAUGACUGUCUUAGGAUGU |
| circLPAR3 shRNA-2 | AUGACUGUCUUAGGAUGUUCA |
| IGF2BP2 shRNA | CATGCCGCATGATTCTTGA |
| PCBP2 shRNA | GCAUUAGCCUGGCUCAAUA |
| CHAC1 shRNA | GACGGGGAAAUUCGAACGC |
| METTL3 shRNA | CTGCAAGTATGTTCACTATGA |
| METTL14 shRNA | CCATGTACTTACAAGCCGATA |
| WTAP shRNA | GGCAAGTACACAGATCTTAAC |
| FTO shRNA | GGTTCACAACCTCGGTTTAGT |
| ALKBH5 shRNA | GCTGCAAGTTCCAGTTCAAGC |
