# Supplementary material for: N6-methyladenosine-modified circLPAR3 drives docetaxel resistance in prostate cancer by suppressing ferroptosis through the PCBP2/CHAC1 axis
Source: Genes Dis. 2025 Aug 22;13(6):101816. doi: 10.1016/j.gendis.2025.101816 (PMC13487614; doi:10.1016/j.gendis.2025.101816)
Supplement: Supplementary file 4 [file mmc4.docx]

**Table S3. Probes used in this study.**

| **Probes name** | **Sequence (5’-3’)** | **Application** |
| --- | --- | --- |
| circLPAR3 probe | GAGAAGTGAACATCCTAAGACAGTC | Pull down |
| control anti-probe | GACTGTCTTAGGATGTTCACTTCTC | Pull down |
| circLPAR3 probe | TGGAGAAGTGAACATCCTAAGACAG | FISH |
