# Supplementary material for: N6-methyladenosine-modified circLPAR3 drives docetaxel resistance in prostate cancer by suppressing ferroptosis through the PCBP2/CHAC1 axis
Source: Genes Dis. 2025 Aug 22;13(6):101816. doi: 10.1016/j.gendis.2025.101816 (PMC13487614; doi:10.1016/j.gendis.2025.101816)
Supplement: Supplementary file 5 [file mmc5.docx]

**Table S4. The antibody information.**

| Antibody Name | Manufacturer | Catalog Number | Application |
| --- | --- | --- | --- |
| anti-IGF2BP2 | Proteintech | 11601-1-AP | IP, WB(1:2000) |
| anti-m6A | Abclonal | A19841 | IP |
| anti-PCBP2 | Abcam | AB184962 | WB(1:1000), IP , IF(1:250) |
| anti-PCBP2 | Abmart | T57300 | WB(1:1000), IP |
| anti-CHAC1 | Abclonal | A15584 | WB(1:900), IHC(1:100) |
| anti-LPAR3 | Proteintech | 19509-1-AP | WB(1:800) |
| anti-β-Actin | Abclonal | AC026 | WB (1:100000) |
| anti-GAPDH | Proteintech | 10494-1-AP | WB (1:5000) |
